# Follistatin Is Induced by Ionizing Radiation and Potentially Predictive of Radiosensitivity in Radiation-Induced Fibrosis Patient Derived Fibroblasts

**DOI:** 10.1371/journal.pone.0077119

**Published:** 2013-10-18

**Authors:** Helen B. Forrester, Alesia Ivashkevich, Michael J. McKay, Trevor Leong, David M. de Kretser, Carl N. Sprung

**Affiliations:** 1 Centre for Innate Immunity and Infectious Diseases, Monash Institute of Medical Research, Monash University, Clayton, Victoria, Australia; 2 North Coast Cancer Institute, Lismore, New South Wales, Australia; 3 Division of Radiation Oncology and Cancer Imaging, Peter MacCallum Cancer Centre, East Melbourne, Victoria, Australia; 4 Centre for Reproduction and Development, Monash Institute of Medical Research, Clayton, Victoria, Australia; 5 Department of Anatomy and Developmental Biology, Monash University, Clayton, Victoria, Australia; University Health Network, Canada

## Abstract

Follistatin is a potent regulator of the inflammatory response and binds to and inhibits activin A action. Activin A is a member of the TGFβ protein superfamily which has regulatory roles in the inflammatory response and in the fibrotic process. Fibrosis can occur following cell injury and cell death induced by agents such as ionizing radiation (IR). IR is used to treat cancer and marked fibrotic response is a normal tissue (non-tumour) consequence in a fraction of patients under the current dose regimes. The discovery and development of a therapeutic to abate fibrosis in these radiosensitive patients would be a major advance for cancer radiotherapy. Likewise, prediction of which patients are susceptible to fibrosis would enable individualization of treatment and provide an opportunity for pre-emptive fibrosis control and better tumour treatment outcomes. The levels of activin A and follistatin were measured in fibroblasts derived from patients who developed severe radiation-induced fibrosis following radiotherapy and compared to fibroblasts from patients who did not. Both follistatin and activin A gene expression levels were increased following IR and the follistatin gene expression level was lower in the fibroblasts from fibrosis patients compared to controls at both basal levels and after IR. The major follistatin transcript variants were found to have a similar response to IR and both were reduced in fibrosis patients. Levels of follistatin and activin A secreted in the fibroblast culture medium also increased in response to IR and the relative follistatin protein levels were significantly lower in the samples derived from fibrosis patients. The decrease in the follistatin levels can lead to an increased bioactivity of activin A and hence may provide a useful measurement to identify patients at risk of a severe fibrotic response to IR. Additionally, follistatin, by its ability to neutralise the actions of activin A may be of value as an anti-fibrotic for radiation induced fibrosis.

## Introduction

### Fibrosis

Fibrosis is a major adverse reaction following RT which can significantly compromise quality of life. It is characterized by excessive collagens, glycosaminoglycans and other components of the extracellular matrix. Fibrosis is a part of normal wound healing process, but when there is persistent or severe injury, or if the fibrotic process is dysregulated it can result in the disease state of fibrosis which can be long term and cause significant pain and death [1]. Fibrosis is tightly associated with chronic inflammatory diseases that require continuous wound response, however, the role that inflammation plays in the maintenance of fibrosis is unclear [2]. One mediator of fibrosis is exposure to ionizing radiation such as that used in radiotherapy. This results from the inflammatory reaction to radiation-induced cell death and damage [3]. During this process myofibroblasts are activated which are responsible for tissue remodelling and produce deposits of collagen and other extracellular matrix components [1], [4], [5], [6]. The severity and duration of the radiation insult can influence if fibrosis occurs and its extent, with differences in response between individuals proposed to be due to both environmental and genetic factors [7], [8], [9]. Consequently, a marked sensitivity to radiation in patients may be due to a profound inflammatory response involving factors that drive the fibrotic process or a reduction in the expression of factors that modulate or block the inflammatory and fibrotic reaction.

#### Molecular factors associated with fibrosis

TGF*β* is known to have a major role in the development of fibrosis as many studies have shown that TGF*β* can induce fibrosis, and the lack of TGF*β* in various experimental models can decrease fibrosis [10], [11], [12], [13]. However, the involvement of TGF*β* is complex as it has a wide range of modulation on different cellular processes in addition to inflammation and wound healing which include proliferation, migration, immunity and carcinogenesis [14]. Therefore, the therapeutic benefit from inhibition of TGF*β* has to be placed in the context of the other cellular signalling that would adversely affect an organism such as that observed experimentally in animal models of TGF*β* deficiencies leading to increased tumour rate and autoimmune disorders [15], [16]. Other members of the TGF*β* superfamily may provide alternatives to factors that directly and extensively affect TGF*β* function. Activin is one such candidate which like TGFβ, acts through serine-threonine kinase trans-membrane receptors and utilises the SMAD signalling network to affect downstream targets [17]. The activins are protein dimers formed by two of three different β-subunits [18]. Activin A is the dimer consisting of two βA subunits which are coded for by the *INHBA* gene [19], [20]. The functional value of activin A is apparent from the 100 percent conserved amino acid sequence between humans and rodents [21], [22]. Activin A stimulates mitosis and collagen production *in vitro* [23], [24], [25] and has also been shown to stimulate human lung fibroblasts to differentiate into myofibroblasts [25], important for generation of extra-cellular matrix (ECM) and fibrosis. Activin A is also implicated in the stimulation of fibrosis in a range of experimental models [26] and this action can be inhibited by its binding protein, follistatin; for instance, in a model (carbon tetrachloride (CCl_4_) -induced) of hepatic fibrosis [27] or bleomycin induced pulmonary fibrosis [28].

### Follistatin

The follistatin (FS) amino acid sequence is ∼97 percent conserved across a range of mammals including humans and rodents [21], [29] and is expressed in many tissue types including endothelium and skin. Through an alternative splicing mechanism involving the last exon, *FST* gene produces two main RNA variants, *FST317* and *FST344*, which after translation are cleaved at the C-terminus and produce the two major protein isoforms, FS288 and FS315. In the circulation, FS315 is found at higher levels than FS288, but, although FS288 is found in serum, it is also bound to heparan sulphate proteoglycans on cell surfaces via positively charged heparin binding sites [30], [31]. Follistatin is a glycoprotein which binds activin A and B with high affinity (50–900 pM), and targets the complex to a lysosomal degradation pathway and thus prevents activin from exerting its biological activities. Follistatin can also bind to other TGFβ superfamily proteins including myostatin (Growth and Differentiation Factor 8 (GDF8)), GDF 9, and a number of Bone Morphogenic Proteins (BMPs 2, 5, 7 & 8), however, with affinities 10-fold lower than that for activin [22]. Follistatin has been used in a number of model systems, including the inhibition of fibrosis, to effectively block the actions of activin. It was also shown that TGFβ, *in vitro*, stimulates lung fibroblasts to produce activin A and that follistatin blocked collagen production induced by TGFβ despite being unable to bind the ligand. This study strongly suggests that the fibrotic actions of TGFβ, at least in part, are achieved through its stimulation of activin A [28].

### Expression of follistatin and activin A after ionizing radiation (IR)

Relatively little is known about the expression of follistatin and activins following ionizing radiation such as that received from cancer radiotherapy. Therefore, the transcription levels of activin A and follistatin in a set of primary fibroblast cells derived from patients who developed fibrosis after radiotherapy were determined. The follistatin and activin A translational product secreted into the medium over time after IR were also investigated. Finally, a comparison of the follistatin and activin A level was made between fibroblasts derived from patients who developed excessive fibrosis after radiotherapy and control patients. We found that follistatin and activin A respond to IR at the transcriptional and translational level, and the samples from patients who developed fibrosis had lower levels of follistatin. Therefore, follistatin is a candidate marker for radiosensitivity and may provide an agent that could be used to protect patients from severe fibrotic responses.

## Materials and Methods

### Ethics statement

All patients have given written informed consent and studies have been approved by the Peter MacCallum Cancer Centre and Monash University Ethics Committees.

### Cell culture

Primary fibroblasts were generated from skin punch biopsies from the thigh (distant from the field of radiotherapy) derived from patients who presented severe fibrosis more than 6 months after RT for breast cancer. These had been classified in the Radiotherapy Oncology Group (RTOG) criteria for radiosensitivity as 3 or 4. Control fibroblasts were derived in the same manner but from cancer patients classified as RTOG 0–1. Fibroblast cells were derived as previously described [32], [33] and grown in DMEM medium supplemented with 15% fetal bovine serum and gentamicin and incubated in a 5% CO_2_ humidified incubator at 37°C. Cells were irradiated with a ^137^Cs source and subsequent RNA isolation was performed at various times after IR as described below.

### RNA Isolation

Cells were grown to 80 percent confluency, trypsinized and resuspended in 3 ml PBS and an equal volume of Trizol (Invitrogen, Carlsbad, CA, USA) was added. The cells were homogenized using an 18 gauge needle, the aqueous layer extracted with equal volume of chloroform, mixed with an equal volume of 70 percent ethanol and added onto a RNeasy column (Qiagen, Venlo, The Netherlands). The RNA extraction was continued by using the RNAeasy method as per manufactures recommendations. RNA concentration and integrity was determined by analysing on a bioanalyzer (Agilent, Santa Clara, CA, USA). RNA was determined to be high enough quality if a minimum RIN of 8.5 was obtained.

### Exon arrays

GeneChip Human Exon 1.0 ST Array analysis was performed as per the ‘GeneChip Whole Transcript (WT) Sense Target labelling assay Manual’ (Affymetrix, Santa Clara, CA, USA) [33], [34]. Twenty-four exon arrays were performed on RNA from primary fibroblasts 4 hours after sham irradiation or exposure to 10 Gy IR for each of the 12 patient samples; 6 derived from patients with severe fibrosis following radiotherapy, and 6 control patients. A ^137^Cs source with a dose rate of 0.62 Gy per minute was used to irradiate the cells. The rRNA from 1 µg of total RNA was reduced using a RiboMinus Human/Mouse Transcriptome Isolation Kit (Invitrogen, Carlsbad, CA, USA). Gene expression was assessed as previously described [33], [34]. The array data used in this paper can be found at the gene expression omnibus database: accession number GSE26841. The following files relate to this manuscript: GSM660490, GSM660491, GSM660494 GSM660498, GSM660506, GSM660507, GSM660509, GSM660510, GSM660512, GSM660516, GSM660523, GSM660524, GSM660526, GSM660527, GSM660529, GSM660530, GSM660533, GSM660537, GSM660545, GSM660549, GSM660556, GSM660557, GSM660559, GSM660560. A paired student's t test was used to obtain p-values from the average expression for each exon of a particular gene.

### Quantitative real-time PCR

Primers were designed to candidate exons or genes using ‘primer 3’ on-line freeware software. The primers were then checked for secondary structure (premier biosoft international) and for uniqueness using NCBI primer blast (ncbi.nih). The following PCR primer pairs were used to amplify *FST* (5′-GCTGAGCACCTCGTGGACCG-3′ and 5′-CAGGGGATGCAGTTGGGGGC-3′), *FST*344 (5′-GTCTGTGCCAGTGACAATGC-3′ and 5′-GTCTTCCGAAATGGAGTTGC-3′), *FST*317 (5′-CAACTGAATCTGCCCGTAAA-3′ and 5′-TTTGTTTTTGGCATCTGCTG-3′) and *INHBA* (5′-GGGCAAAGTCGGGGAGAACGG-3′ and 5′-CCTGGCTGTTCCTGACTCGGC-3′). *PGK* (5′-CTGGAGAACCTCCGCTTTCAT-3′ and 5′-TGGCTCGGCTTTAACCTTGTT-3′) was used as a reference for quantitative real time PCR (qRT-PCR). cDNA was made from 1 µg RNA (same RNA as used in the exon arrays) by initially heating to 65°C for 5 minutes and then mixing with 5x first strand buffer (Invitrogen, Carlsbad, USA), 0.1 M dithiothreitol, 0.5 mM deoxynucleotriphosphates and 250 ng of random hexamers and heating to 25°C for 5 minutes with a subsequent incubation of 50°C for 1 hour and a 70°C incubation for 15 minutes. PCR amplification was carried out using 1.25 Units Go-Taq polymerase (Promega, Wisconsin, USA), 200 nM primers, 5 ng cDNA, with a cycling protocol of 95°C: 2 min; ((95°C: 15 sec; 60°C: 45sec; 72°C: 30 sec) ×30); 72°C: 5 min. Products were run on a two percent agarose gel to verify the predicted size for the amplified product. Real-time PCR was performed using these primers under the following conditions. Sybr Green Master Mix (Applied Biosystems, United Kingdom) was mixed with 5 to10 ng of cDNA. The cycling steps were as follows. 95°C: 10 min; ((95°C: 15 sec; 60°C: 60 sec) ×40 with a melting temperature ramp following amplification. A robotic system was used to load a 384 well plate with a subsequent run on the ABI 7900 quantitative real time PCR machine. All samples were run in triplicate on each plate. qRT-PCR was run at least 3 separate times for each patient's fibroblast sample.

### Measurement of follistatin and activin A

4×10^5^ cells were plated in duplicate into 25 cm^2^ flasks and cultured for 48 hrs. All cells were in log phase growth. Medium was replaced with 6 ml of 10 percent fetal bovine serum, antibiotics-free DMEM medium 24 hrs prior to treatment. Cell culture medium from IR-treated or untreated cells was collected at 8, 24, 48, and 72 hrs post-IR, and centrifuged for 20 min at 1500 rpm. The supernatant was immediately frozen. Cells were trypsinized and counted. Cell number and proportion of dead cells was determined using trypan blue (0.1% (w/v)) exclusion method. To test if cell density affects follistatin secretion, fibroblasts were plated at different densities. Levels of follistatin were measured by specific discontinuous radioimmunoassay as described previously [35]. The level of follistatin in medium not exposed to cells was less than 1.03 ng/ml and the average intra-assay coefficient of variance was 8.23 percent. Levels of activin A were measured by ELISA specific for activin β_A_ subunit [36]. The activin A content in medium was less than 11.95 pg/ml and the average intra-assay coefficient of variance was 8 percent.

### Next Generation sequencing

RNA from fibroblast samples, with or without 10 Gy IR treatment (four samples for sequencing), were sequenced by Yourgene Bioscience (New Taipei City, Taiwan) using an Illumina platform under the CSPro program. Bioanalysis RNA integrity numbers for all samples used were 10. A quality trim was performed after paired-end RNA sequencing which eliminated reads with a poor overall Phred score and trailing bases with poor quality. Total base reads after a quality trim exceeded 4.3×10^9^ per sample. The average read length after a quality trim was 95 bases. BAM files were provided from Yourgene Bioscience and uploaded to Partek Genomics Suite 6.6 (St Louis, Missouri, United States) RNA-Seq analysis program or Integrative Genomics Viewer 2.0 for graphic representations of analysis.

## Results

### Radiation induces follistatin and INHBA

**Figure 1:**
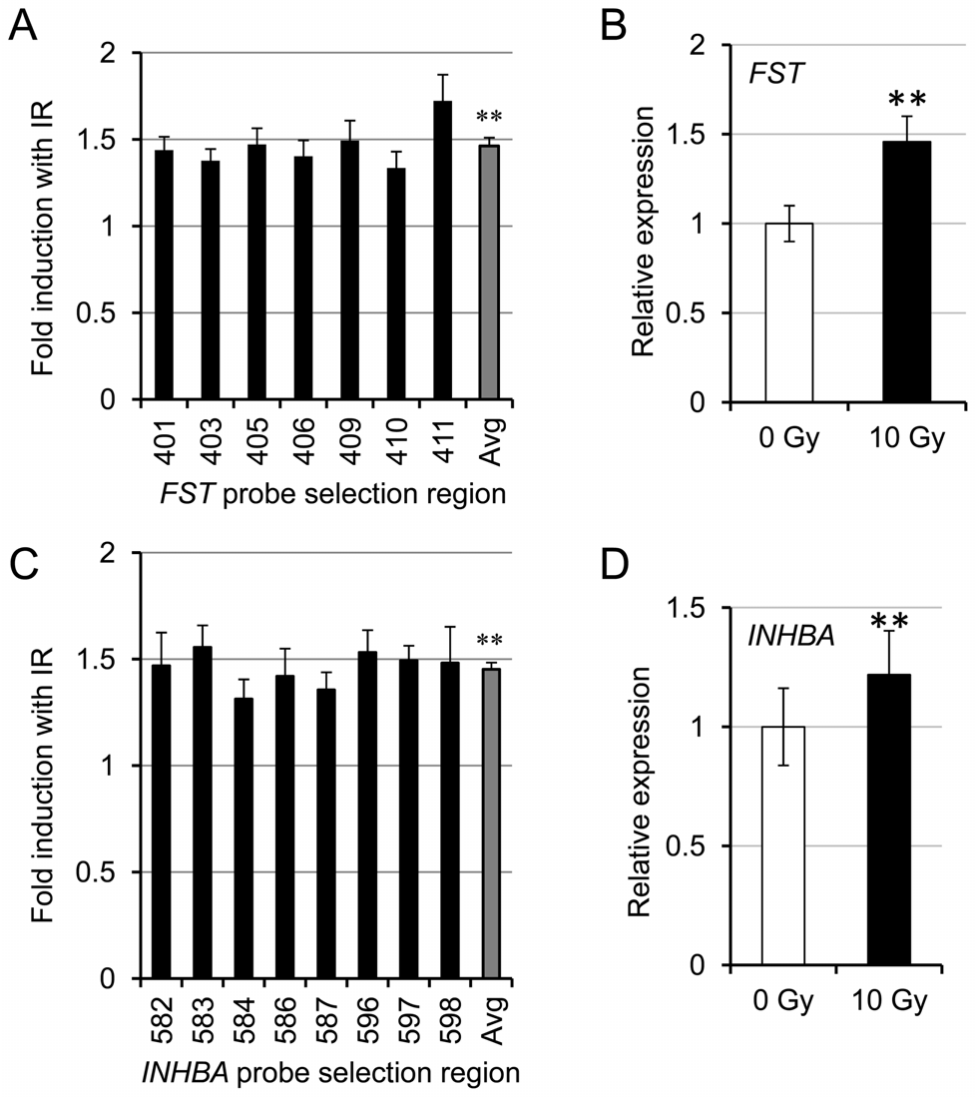
IR induces *FST* and *INHBA* gene expression. (A) Exon array expression data is plotted for each of the seven *FST* exons. The fold induction of *FST* after IR for each exon (black bars) and for the gene average (grey bar) are shown. The data have been derived from 24 individual exon arrays (n = 12 different patient's fibroblasts for both 0 Gy and 10 Gy). The last three digits of the probe selection regions, which correspond to exons, are indicated below the bar graph. (B) qRT-PCR validation for *FST* gene expression after IR (n = 5 utilizing the same control patient's fibroblasts as used for the exon arrays; p<0.005). (C) Exon array expression data is plotted for each of the eight *INHBA* exons as in (A) (n = 12). The PSRs are indicated below the bar graph (D) qRT-PCR validation for *INHBA* gene expression after IR (n = 5 as for (B); p<0.01). Error bars represent SEM. Student's paired t-test was used to show statistical significance. Asterisks (∗∗p<0.01) indicate statistical significance between 0 Gy and 10 Gy treated samples.

We interrogated follistatin and activin genes on the exon level in response to 10 Gy of IR four hours post-IR [33], [34]. For IR response experiments, 12 exon array chips were hybridized for each dose (total  = 24 microarrays) where a different exon array was used for each individual sample and condition from which expression data was generated. There are multiple probes for each of 7 exons for the *FST* gene and 8 exons for the *INHBA* gene. The *FST* gene showed a transcriptional up-regulation in response to radiation at 4 hr post-IR (Figure 1A) which for the whole gene was statistically significantly different (p<0.01). *FST* had a relatively high basal expression level, so this is a substantial increase in number of transcripts. To confirm these results, the *FST* response to IR was assessed using qRT-PCR (Figure 1B; p<0.01). *FST* gene was found to be induced after 10 Gy IR by approximately 1.5 fold. Expression levels of the activin A gene (*INHBA*) was also increased as determined from the exon arrays (Figure 1C; p<0.01) and these results were validated with qRT-PCR (Figure 1D; p<0.01). *INHBA* had a relatively high level of inter-individual variation; however, its gene expression was increased 4 hours after 10 Gy of IR treatment in every case (Figure S1).

### Measurement of follistatin isoforms

**Figure 2:**
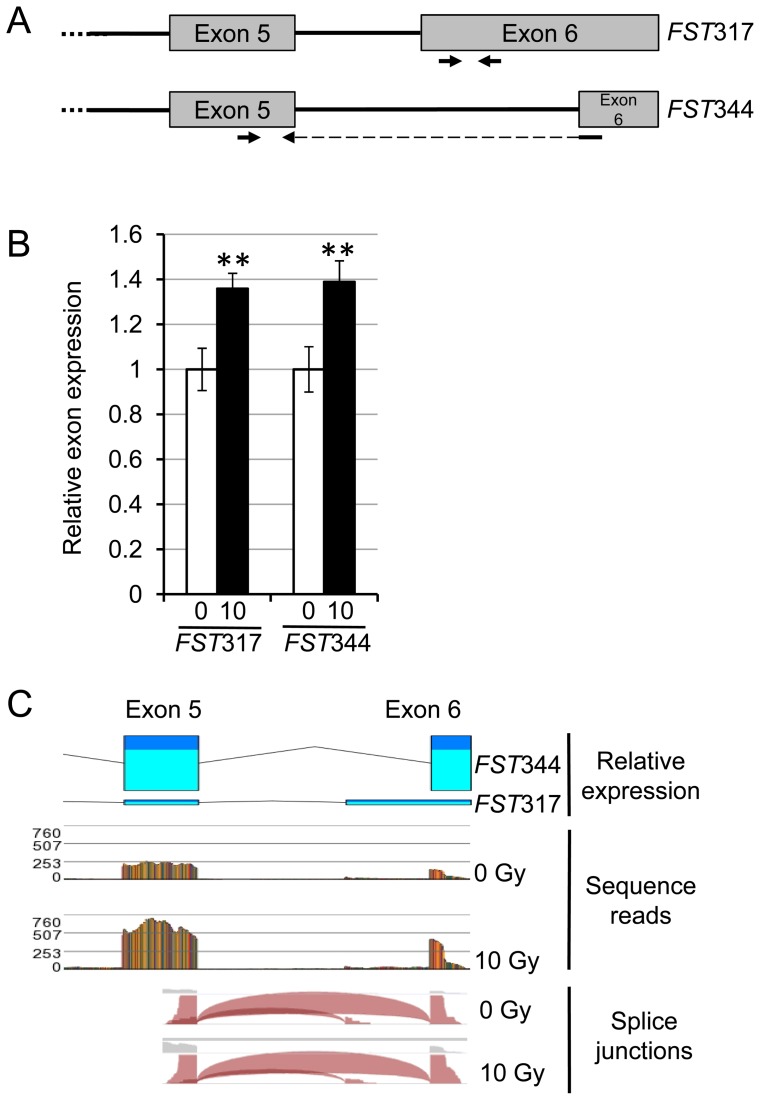
The two major *FST* transcript variants are induced by radiation. (A) A schematic shows the gene structure in the last 2 exons for the two major *FST* variants with the positioning of variant specific primers indicated (arrows). (B) qRT-PCR analysis of the two *FST* transcript variants, *FST317* and *FST344*, from primary fibroblasts before (white bars) and 4 hours after IR (black bars) (n = 5 different control patients' fibroblasts). A paired t-test was used to show a statistically significant difference in the transcription levels for both *FST* variants 4 hours after 10 Gy of IR (p<0.005). (C) RNA-Seq data of the *FST* gene showing the transcript variants from a fibroblast (from a control patient (C4)) treated with or without IR. The two transcript variants can be seen in panel C (top), showing the *FST*344 has many more sequence reads as indicated by the taller exon bar (normalized to the sequence read total number). Total quality trimmed base reads for 0 Gy and 10 Gy treated samples were 4.6×10^9^ and 4.9×10^9^ bases respectively. Both basal (dark blue) and irradiated levels (light blue) of the two variants can be visualized. Junctions between exons are indicated at bottom of panel. Asterisks (∗∗p<0.01) indicate statistical significance between 0 Gy and 10 Gy treated samples. Error bars represent SEM.

Follistatin is known to have two major transcript variants [37], [38], *FST*317 and *FST*344. The variants have different properties in that the *FST*344 product results in a circulation form and the product from the *FST*317 results in a heparin/heparan sulphate binding form and, therefore, it was of interest to evaluate the relative expression kinetics in response to IR. The transcript levels of both *FST* alternative transcripts were assessed by qRT-PCR utilizing primers that are able to specifically amplify the different transcript variants (Figure 2A) which was confirmed by sequencing the amplicons. Both transcript variants showed an increase in gene expression 4 hours post-IR following treatment with 10 Gy IR (Figure 2B, p<0.005) and the increase after IR is approximately the same for both variants. qRT-PCR indicated that *FST*344 variants were expressed at higher levels than the *FST*317 variant. RNA-Seq confirmed this observation showing that there was approximately ten times more *FST*344 than *FST*317 variant (Figure 2C) but the fold-increase of induction specific for each variant after IR was similar (Figures 2B and 2C).

### FST and INHBA transcription levels in radiosensitive fibrosis patients

**Figure 3:**
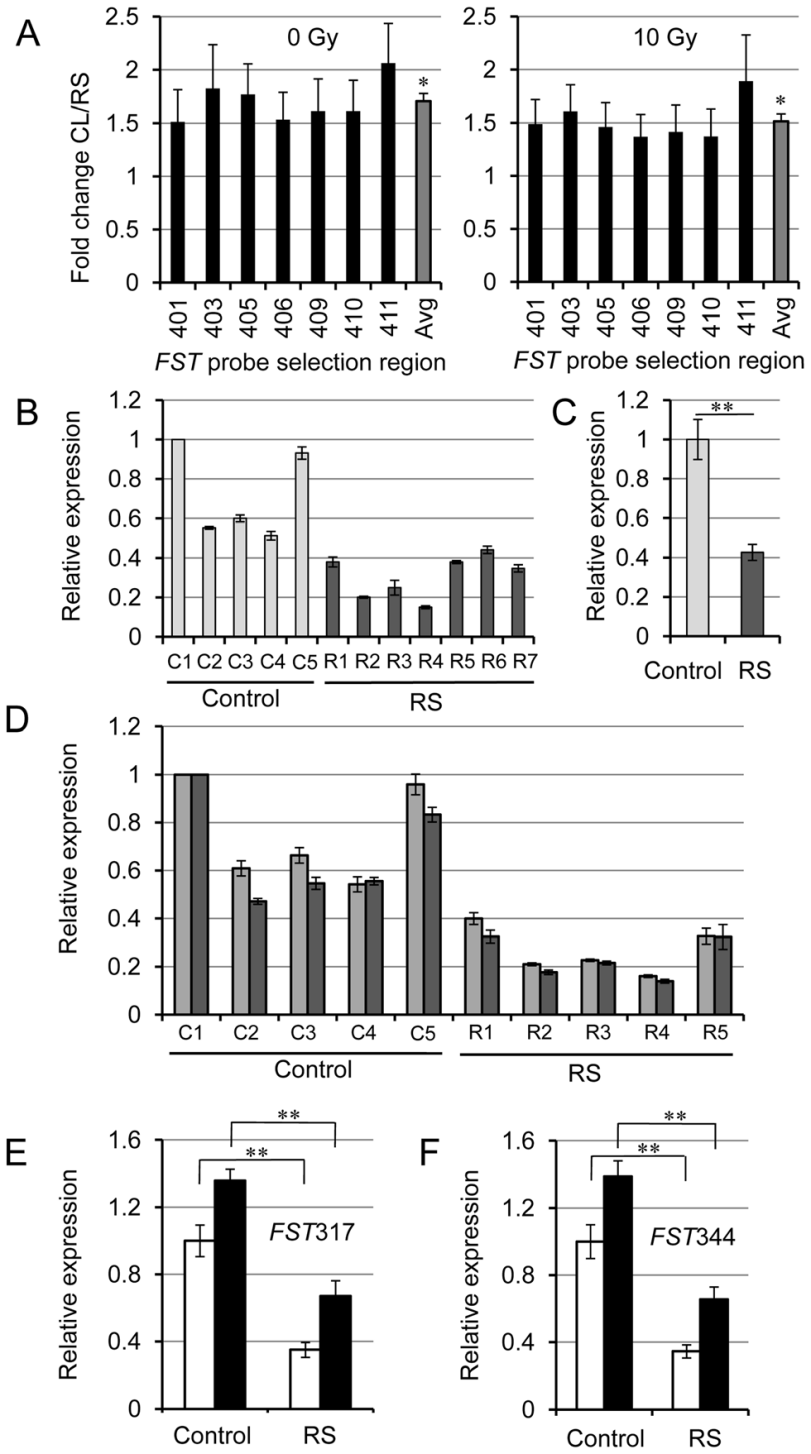
*FST* gene expression is reduced in fibrosis patient fibroblasts. (A) Exon array expression data is plotted for each of the seven *FST* exons before (0 Gy: left panel) and after IR (10 Gy: right panel). The last three digits of the probe selection regions, which correspond to exons, are indicated below the bar graph. The fold expression change (control/fibrosis samples) for exons (black bars) and the gene average (grey bar) are shown. (n = 6 different patient's fibroblasts from individual exon arrays for each patient cohort). (B) qRT-PCR data showing basal level gene expression of FST from 12 separate samples from different radiotherapy patients; seven who had radiosensitivity (Dark grey bars; radiosensitive: R1 to R7) and five who did not elicit fibrotic symptoms (Light grey bars; control: C1 to C5). (C) The average basal levels for *FST* transcripts from panel B are plotted. There is a statistically significant difference between the control group and in the radiosensitive group FST levels (p = 0.002). (D) qRT-PCR data showing basal level gene expression of *FST*317 (light grey) or *FST*344 (dark grey) *FST* variants in cells from 10 separate samples from different patients; five who had fibrosis (radiosensitive: R1 to R5) and five who did not elicit fibrotic symptoms (control: C1 to C5) after radiotherapy. The error bars represent the SEM, and the expression levels obtained were derived from three to eight different qRT-PCR experiments on the same RNA. The relative basal expression levels in the radiosensitive patients are all lower than the levels in the control patients for both *FST* transcript variants (p<0.005). (E) Both basal levels (open bars) and levels 4 hours after 10 Gy IR (black bars) for *FST317* transcripts were tested (n = 5 patient's fibroblasts). There was a significant difference after irradiation both in the control group (p<0.005) and in the radiosensitive group (p<0.001). There is also a lower level of *FST317* expression after IR in the radiosensitive patients compared with the control patients at both the basal level and after IR (p<0.005). (F). qRT-PCR analysis of the *FST*344 transcript variant levels as shown for *FST*317 in (E). The results were similar for both the transcript variants. Statistical significance was determined using a Student's t-test. Asterisks (∗∗p<0.01) indicate statistical significance between control and radiosensitive/fibrosis samples. Error bars represent SEM.

A comparison of *FST* or *INHBA* gene expression in fibroblasts between patients who had high levels of fibrosis following IR and those who were non-symptomatic was conducted. Exon array analysis showed that *FST* transcript levels were lower in fibrosis patient samples compared to controls at basal levels and 4 hours after 10 Gy IR treatments (Figure 3A). Seven samples from the radiation response group and five samples from the control group were used to test the FST gene expression using qRT-PCR (Figure 3B). The difference between the controls and radiosensitive groups were statistically significant (Figure 3C; p = 0.002). The response of the specific transcript variants was also assessed (Figures 3D–E). Five samples from each radiation response group (controls and radiosensitive fibrosis patients) were run and the ratio of transcription levels of the two different variants within individual samples were similar (Figure 3D). Both transcript variants for *FST* were statistically significantly lower (p<0.005) in fibroblasts derived from the patients with an excessive fibrotic response compared to the non-symptomatic controls both before and after irradiation (Figures 3E and 3F; The response for individual patients is shown in Figure S2.). The *FST* expression levels were lower in all the fibrosis patients compared to all the controls (Figures 3B and 3D). Similar analyses were performed for *INHBA* and *INHBB* gene transcripts which code for activin A and B, respectively, but they did not show significant modulation between radiosensitive and control patient samples (Figures S1 and S3). Also, *INHBB* did not show any modulation after IR (Figure S3).

### Follistatin and activin A protein response after IR

**Figure 4:**
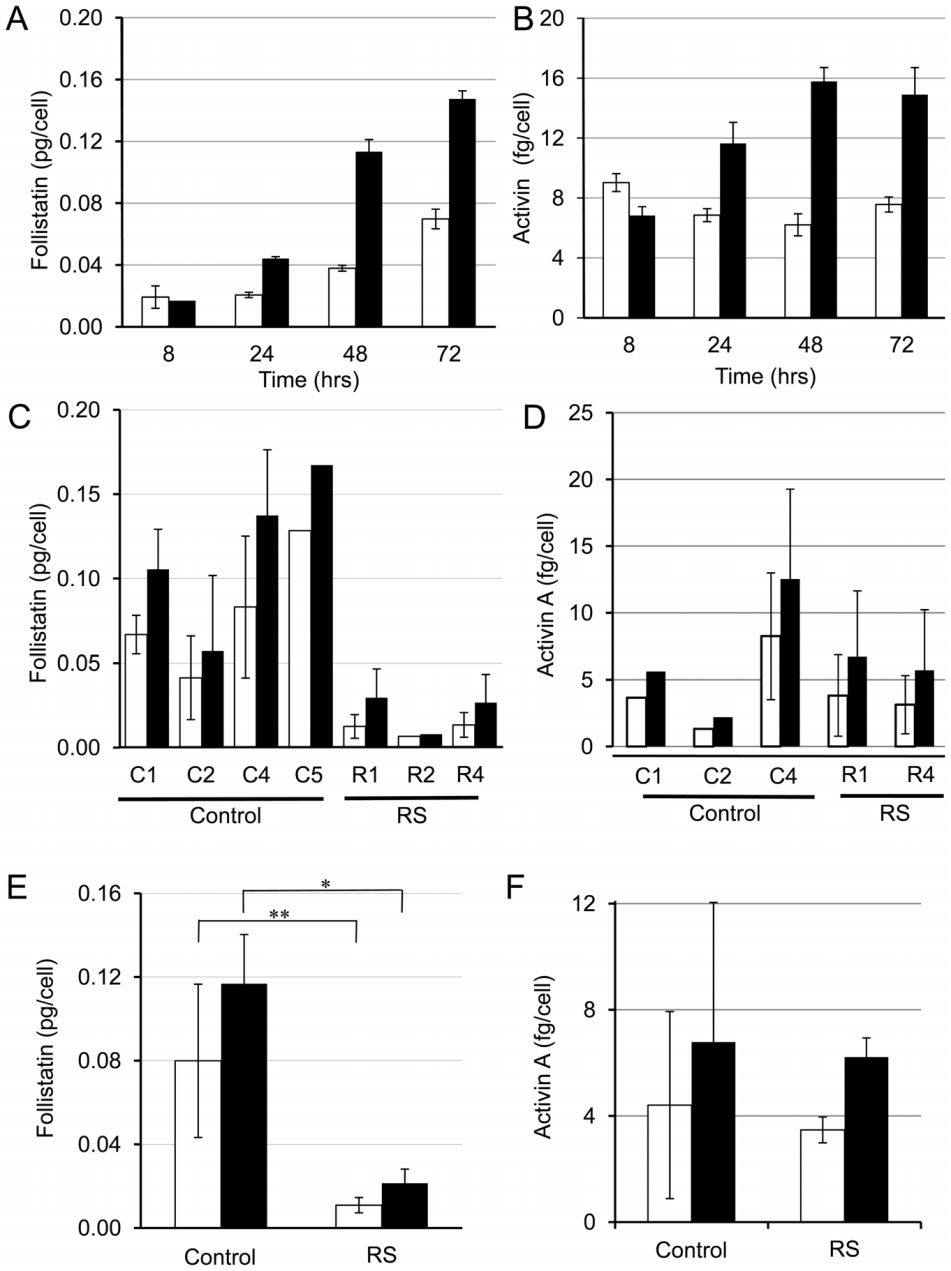
Secreted follistatin and activin A β_A_ subunit protein levels are induced in response to IR and follistatin predicts radiosensitivity before and after IR. Primary fibroblasts were treated with (black bars) or without (open bars) 10 Gy IR. The levels of follistatin or activin A in the medium of fibroblasts were determined by RIA and ELISA, respectively. Follistatin (A) or activin A (B) protein levels secreted into the medium were measured over a time course in fibroblasts. The mean ± SD of two separate RIA assays (experimental replicates) are shown for sample R1 (A, B). (C–F) Samples were collected 24 hours after IR. The mean ± SD of at least 2 experiments except for C5 and R2 in panel C; and C1, C2 in panel D where one experiment was performed (no error bars). The production of follistatin (C, E) or activin A (D, F) was measured as described in the Materials and methods. Panels E and F show the averages of the tested samples from C and D, respectively. A Student's paired t-test indicated a significant induction of follistatin (C; ∗p = 0.032) and Activin A (D; ∗p = 0.016). Error bars for panel E and F represent the SD (for (E) n = 3 (fibrosis patients' fibroblasts) or n = 4 (control patients' fibroblasts) and for (F) n = 2 (fibrosis patients) or 3 (control patients)). Production of follistatin was significantly different between controls and radiosensitive patients at a basal level (∗∗p<0.01) and after IR (∗p<0.05). Activin A levels were not significantly different between control and radiosensitive groups (p = 0.856).

The response at the transcription level is commonly associated with the translational response; therefore, we investigated whether follistatin and activin protein levels were modulated by IR. Fibroblast cultures were irradiated with 10 Gy and medium was harvested at different time points after IR. Trypan blue exclusion indicated that even after 72 hours post IR very little cell death was observed (<3%). Ionizing radiation can cause cell cycle arrest [39], [40] and inhibited cell proliferation (Figure S4). To account for this, protein levels were normalized to cell number. To test if cell density effects follistatin secretion, we plated cells at different densities and found that the secretion of follistatin in response to IR was not influenced by cell density (Figure S5). RIA showed that follistatin protein in fibroblast medium was increased at 24, 48 and 72 hours after IR but not at the earliest 8 hour time point (Figure 4A). ELISA indicated that activin A (β_A_ subunit) protein was also induced after IR, but not at the 8 hour time point (Figure 4B). The follistatin levels in the medium of cell cultures from the patients who had an excessive fibrotic response were significantly lower than controls both prior to, and after irradiation (Figures 4C and 4E; p<0.05). The medium concentrations of activin A also showed a rise but this change after IR was not statistically significant (Figures 4D and 4F).

## Discussion

Follistatin can neutralize a number of TGFβ superfamily members and has been especially prominent in the regulation of activins [19], [41]. Activin A induces the expression of fibrotic control regulators and can stimulate fibroblasts to differentiate into myofibroblasts, key in fibrosis [19]. The present studies have found that follistatin and *INHBA* (activin β_A_ subunit transcript) gene expression is induced in response to IR which is also reflected at the translational level. It is of interest that radiation directly induces changes in fibroblasts and the response does not require the influx of inflammatory cells. We also found that the levels of follistatin gene expression and protein secretion in fibroblasts were able to differentiate most of our radiation induced fibrosis patients from non-fibrosis patients. These data suggest that follistatin stands out as a potential therapeutic to modulate fibrosis since *in vitro*, it is both radio-responsive and is differentially expressed between fibrosis and control samples. The lower follistatin expression level in samples from radiosensitive patients is consistent with a decreased ability of follistatin to block the actions of activin A thereby contributing to increased or excessive fibrotic response.

These data are also consistent with other studies which have looked at follistatin treatment of chemically induced fibrosis in animal models and have demonstrated its therapeutic potential. For example, follistatin was found to reduce fibrosis in a bleomycin treated pulmonary fibrosis mouse model [28]. In these studies it was found that infiltration of inflammatory cells such as macrophages and neutrophils were reduced with follistatin treatment as were the proinflammatory cytokines IL-1B and MCP-1/CCL2. They also found a reduction in hydroxyproline content, a measure of fibrosis which was complemented by histological staining. They concluded this was accomplished by blocking the actions of activin and TGFβ. In a separate animal model, follistatin attenuated fibrosis in CCl_4_-induced hepatic fibrosis as determined by histochemical staining and by hydroxyproline content [27]. It will be of interest in the future to test the effect of activin A inhibitors such as follistatin as an antifibrotic for radiation-induced fibrosis *in vivo*.

There are a number of examples where treatments for radiation-induced fibrosis have shown promising results. Halofuginone inhibits various members of the TGFβ signalling pathway [42], [43] and reduces the synthesis of type I collagen [44] and phosphorylation of SMAD3 [45]. In an animal model, halofuginone caused a significant decrease in fibrosis in radiation-treated mice [42]. However, side effects, such as bleeding, nausea, vomiting and fatigue, have been reported together with high inter-patient variability in responses [46]. Pirfenidone and other broad spectrum compounds, that modulate the TGFβ superfamily show promise for fibrosis treatment [47], [48], [49], [50], [51]. However, the long term efficacy and safety has recently been questioned [52], [53]. Also, treatment with an anti-TGFβ1 antibody resulted in a reduction of ECM synthesis and fibrosis in irradiated tissues in a rat model [54]. Activin provides another very promising target for anti-fibrotics given its role in cross talk with TGFβ and utilizing some of the same downstream signalling pathways, but also having distinct actions and mediators from TGFβ.

Two major *FST* transcript variants responded similarly to radiation with an increase in expression levels after IR, but the *FST344* was present at much higher levels. The *FST344* variant codes for the circulating longer protein isoform FS315, and *FST317* variant codes for the FS288 protein which is known to bind heparan sulphate proteoglycans on cell surfaces. The heparin binding site in FS315 is masked by a protein tail which becomes exposed after the FS315 binds to activin enabling binding to the heparin/heparan sulphate on the cell surface. Both follistatin isoforms are capable of inactivating activin, but FS288 has been shown to have a considerably longer duration of action in lowering serum follicle stimulating hormone levels when injected intravenously into ovariectomised rats [55] thus favouring FS288 as a preferred therapeutic. The longer duration of action may be due to the capacity of FS288 to bind to heparan sulphate proteoglycans and retain its bioactivity whereas the inability of FS315 to bind in a similar manner may render it liable to be cleared through degradation pathways. Such issues will need to be considered in therapeutic applications.

In addition to activins, follistatin also inhibits other members of the TGFβ superfamily such as BMPs. BMP family members are involved in the control of cell growth and differentiation and are also involved in fibrosis. BMP7 inhibits fibrosis in the kidney [56] and liver [57], however, the BMP, myostatin is possibly profibrotic stimulating muscle fibroblasts to proliferate [58]. Myostatin possibly increases resistance of fibroblasts to apoptosis through SMAD and MAPK signalling and inhibition of myostatin may increase apoptosis in the fibroblasts [58]. In acute muscle injury, fibroblasts are usually activated to proliferate and produce ECM. Once the injury is resolved the activated fibroblasts (myofibroblasts) normally undergo apoptosis [6]. In fibrosis patients the decrease in follistatin may result in an increase in activity of activin A and myostatin possibly suppressing the apoptosis that occurs in myofibroblasts after IR-induced damage has been repaired. In the fibrosis patients the injury repair process may continue, resulting in fibrosis.

Our finding that follistatin at both the transcription and translation levels predicted fibrosis in most of our fibrosis patients provides a good candidate for predicting patient fibrosis susceptibility in the clinic. There have been many attempts to identify a radiosensitivity predictive marker utilizing a variety of assays with limited success [59], [60], [61], [62], [63], [64], [65], [66]. A combination of these markers may enable the development of a clinically applicable predictor and enable radiotherapy regime individualization. There is likely to be a variety of reasons why these patients develop fibrosis. However, our data indicate that follistatin levels may be indicative for a high proportion of these patients. It is probable that not all the radiosensitive patients will be identified by follistatin expression alone due to inter-patient variability and the relatively small differences in expression levels. Therefore, a combination of markers would be more effective to reliable predict radiosensitivity in all patients.

Preclinical studies will be able to inform the optimal timing for treatment. If FS administration prior to or during radiotherapy proves to be effective in preclinical models, the translation to human studies could be rapid since follistatin is presently being tested as a therapeutic in clinical trials for other disease models, including osteoporosis and muscle wasting disease [67].

## Conclusion

This paper provides evidence that the levels of follistatin gene and protein expression could potentially be used to predict the sensitivity of patients to develop radiation-induced fibrosis. Should this data be confirmed in larger studies, it would enable the optimization of radiation dosage for individual patients. In addition to prevent the significant disability that accompanies radiation-induced fibrosis, the ability to use higher doses of radiation in non-sensitive patients would improve therapeutic efficacy. Furthermore, given the capacity of follistatin to suppress the inflammatory and fibrotic responses, patients could be given follistatin to suppress radiation-induced fibrosis.

## Supporting Information

Figure S1 *INHBA* gene expression shows no significant difference between controls and fibrosis patient derived samples before and after exposure to IR. (A) Exon array gene expression data is plotted for each of the 8 *INHBA* exons for control (grey bars) compared to fibrosis (black bars) patient samples (n = 6 different patient's fibroblasts). The averages of all eight exons are shown at the right. Gene expression levels as determined by qRT-PCR for 5 individual control and 5 fibrosis patients (B) before (open bar) and 4 hours after 10 Gy of IR (black bars) for *INHBA*, and (C) the average for control and radiosensitive patients. Error bars represents SEM.
(TIF)

Figure S2 *FST* variant gene expression levels as assessed by qRT-PCR for individual control and fibrosis/radiosensitive patients before and after IR. Gene expression levels for individual control and radiosensitive patients before (open bars) and 4 hours after 10 Gy (black bars) for *FST* variants (A) *FST*317 and (B) *FST*344. Error bars represents SEM from at least three separate qRT-PCR runs.
(TIF)

Figure S3 IR does not induce *INHBB* gene expression and no difference in expression is observed between controls and fibrosis patient derived samples. Exon array gene expression data is plotted for each of the five *INHBB* exons for (A) sham-irradiated (grey bars) and irradiated (black bars) samples (n = 6), and (B) control (grey bars) compared to fibrosis (black bars) patient samples (n = 6). The averages of all five exons are shown at the far right in the bar graphs.
(TIF)

Figure S4 Growth curve of a primary fibroblast cell line and associated follistatin levels. Cell number (A) and follistatin levels in the medium (B) of the R1 primary fibroblasts at time points after IR were determined as described in Materials and Methods. 10 Gy IR-treated (black bars) or sham-irradiated (open bars) are shown.
(TIF)

Figure S5 The effect of cell density on follistatin released into the medium after IR treatment. Cells were plated in 25 cm^2^ flasks at three densities and treated with 10 Gy IR (black bars) or sham irradiated (open bars). Twenty-four hours after IR treatment, cells were counted (A), follistatin was measured in the medium (B) as described in the Materials and methods, and normalized to cell number (C).
(TIF)
