# Supplementary figures and images for: Follistatin Is Induced by Ionizing Radiation and Potentially Predictive of Radiosensitivity in Radiation-Induced Fibrosis Patient Derived Fibroblasts
Source: PLoS One. 2013 Oct 18;8(10):e77119. doi: 10.1371/journal.pone.0077119 (PMC3799767; doi:10.1371/journal.pone.0077119)

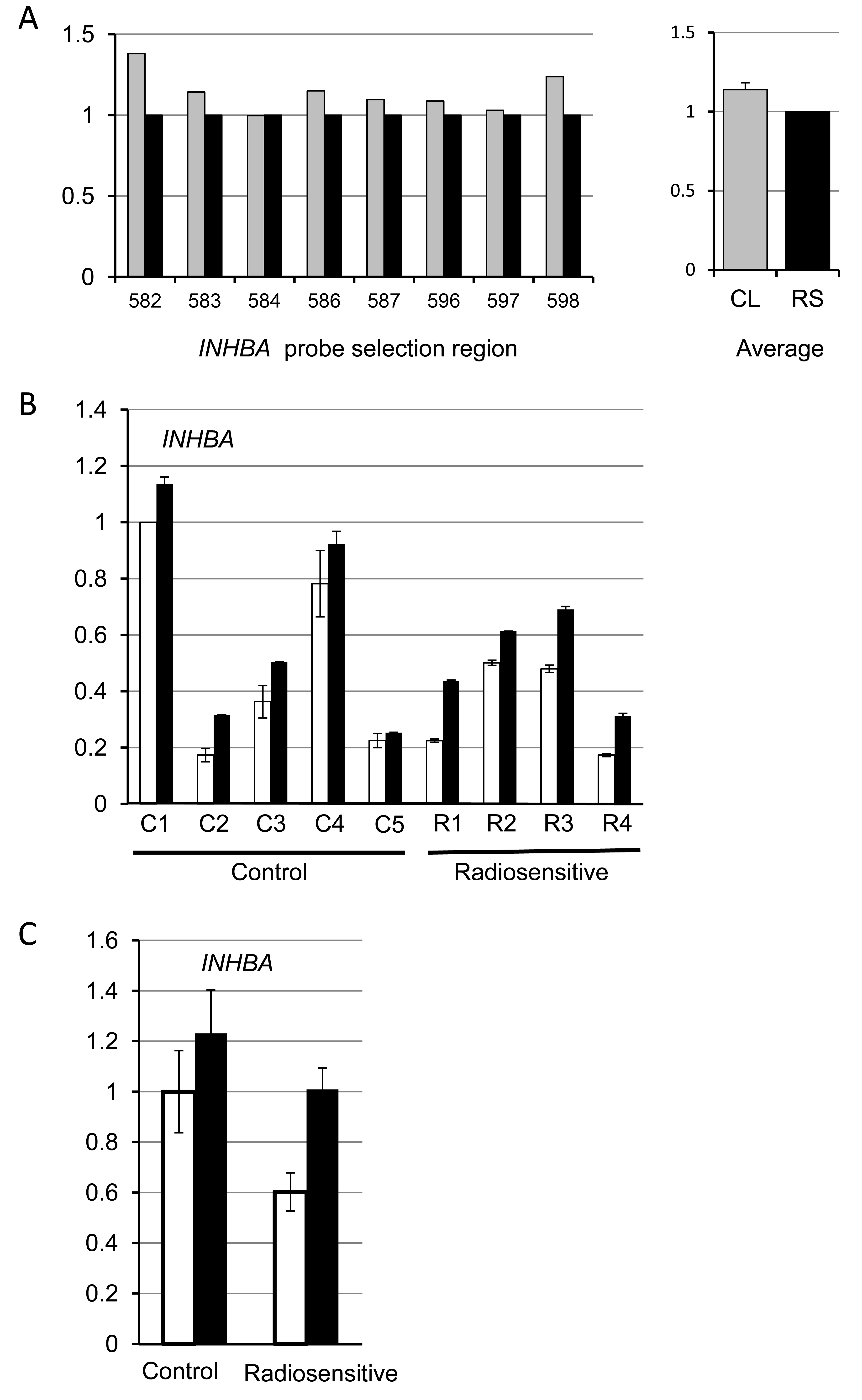

Supplement: Figure S1 — INHBA gene expression shows no significant difference between controls and fibrosis patient derived samples before and after exposure to IR. (A) Exon array gene expression data is plotted for each of the 8 INHBA exons for control (grey bars) compared to fibrosis (black bars) patient samples (n = 6 different patient's fibroblasts). The averages of all eight exons are shown at the right. Gene expression levels as determined by qRT-PCR for 5 individual control and 5 fibrosis patients (B) before (open bar) and 4 hours after 10 Gy of IR (black bars) for INHBA, and (C) the average for control and radiosensitive patients. Error bars represents SEM. (TIF) [file pone.0077119.s001.tif]

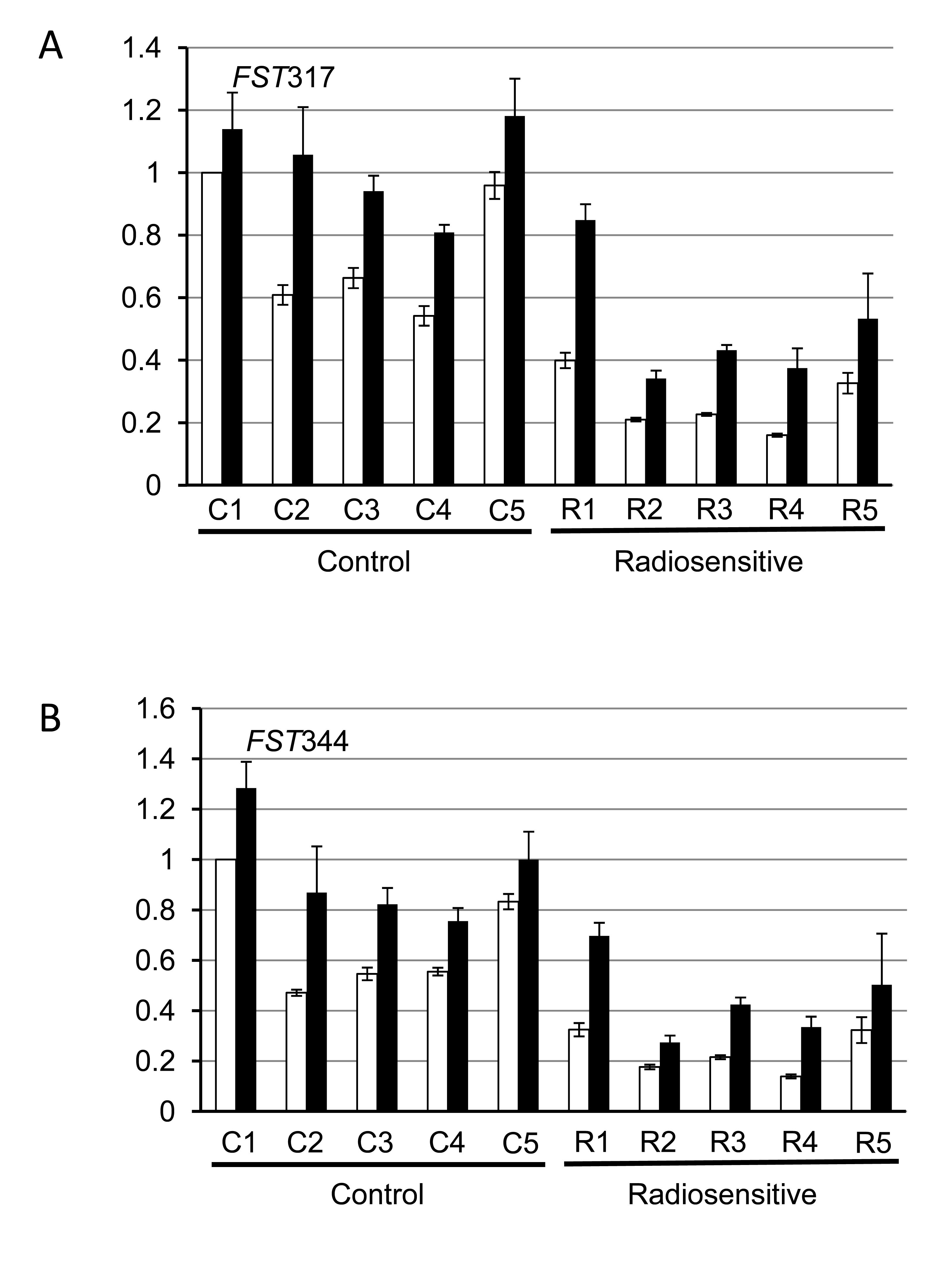

Supplement: Figure S2 — FST variant gene expression levels as assessed by qRT-PCR for individual control and fibrosis/radiosensitive patients before and after IR. Gene expression levels for individual control and radiosensitive patients before (open bars) and 4 hours after 10 Gy (black bars) for FST variants (A) FST317 and (B) FST344. Error bars represents SEM from at least three separate qRT-PCR runs. (TIF) [file pone.0077119.s002.tif]

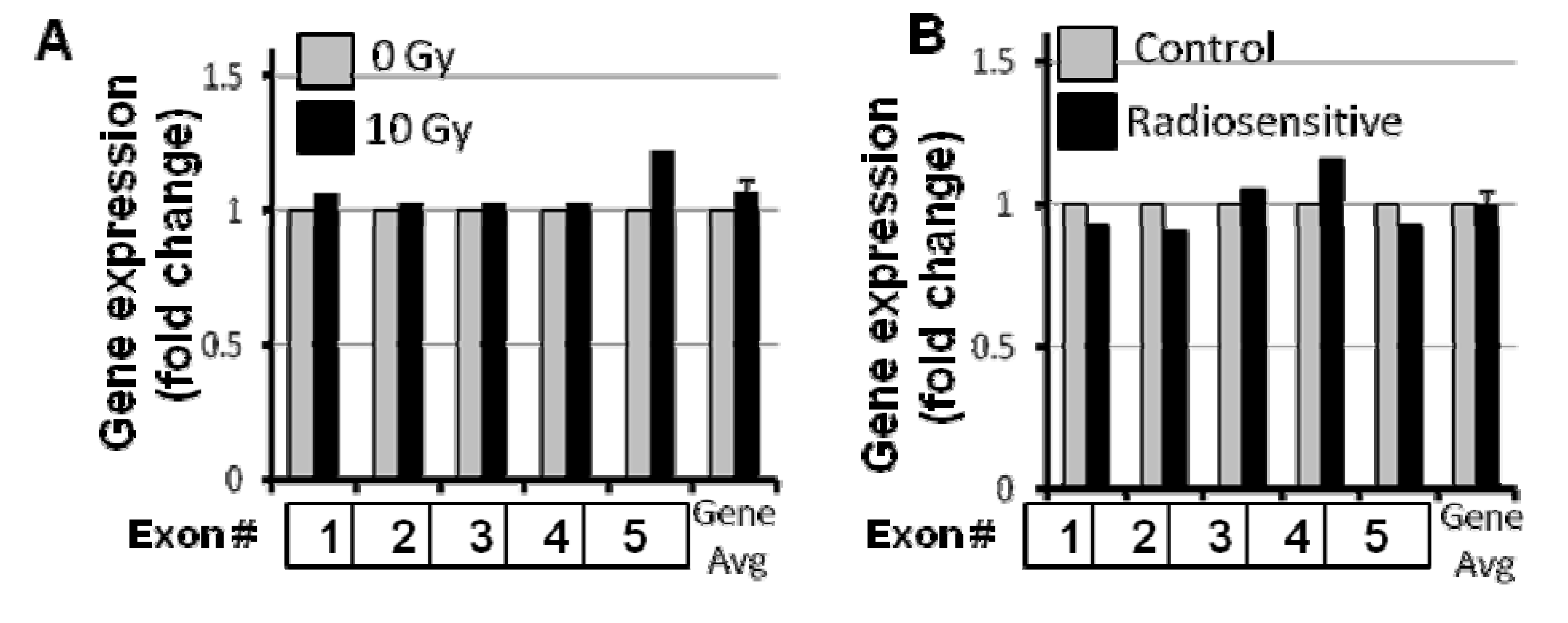

Supplement: Figure S3 — IR does not induce INHBB gene expression and no difference in expression is observed between controls and fibrosis patient derived samples. Exon array gene expression data is plotted for each of the five INHBB exons for (A) sham-irradiated (grey bars) and irradiated (black bars) samples (n = 6), and (B) control (grey bars) compared to fibrosis (black bars) patient samples (n = 6). The averages of all five exons are shown at the far right in the bar graphs. (TIF) [file pone.0077119.s003.tif]

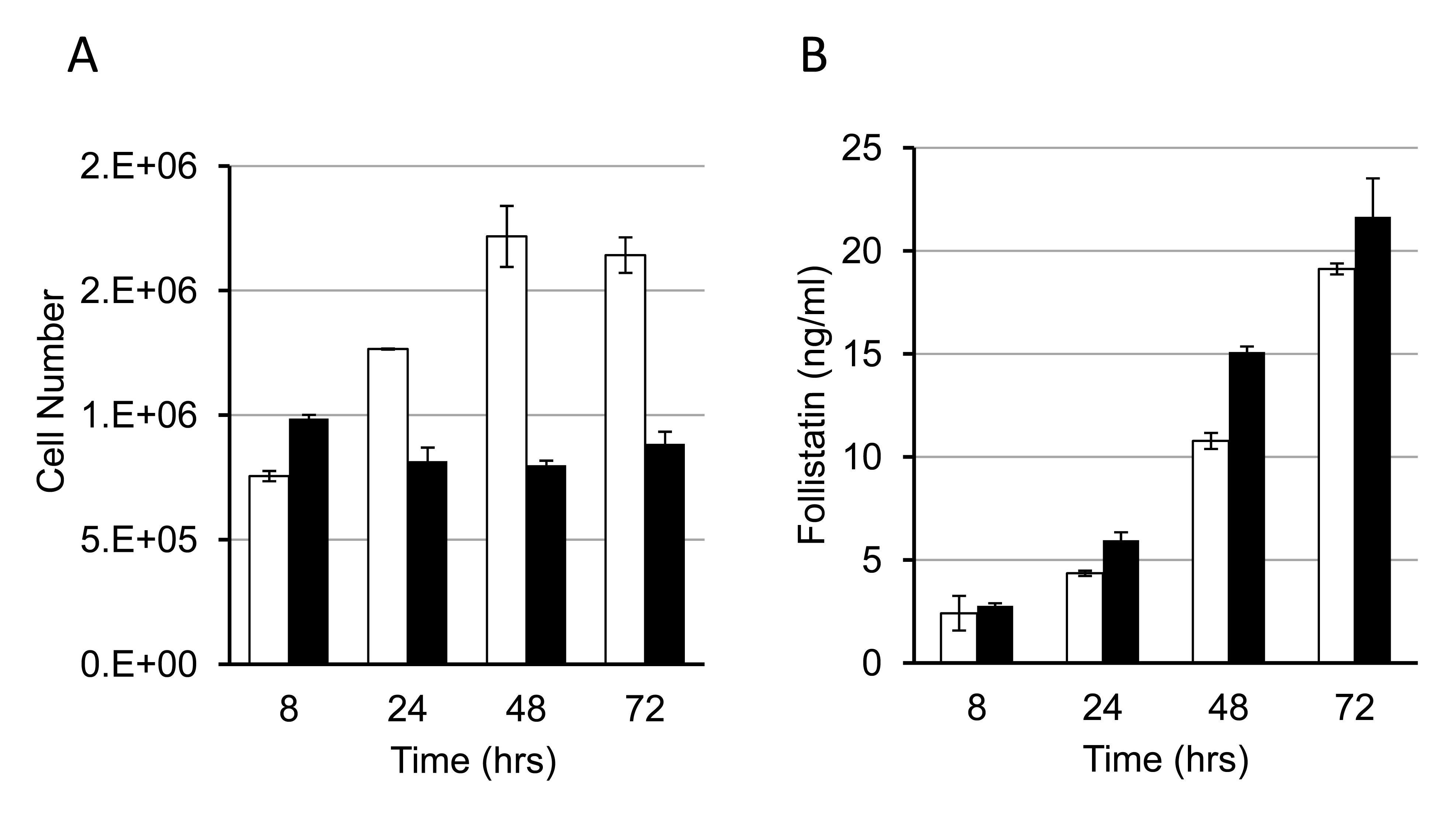

Supplement: Figure S4 — Growth curve of a primary fibroblast cell line and associated follistatin levels. Cell number (A) and follistatin levels in the medium (B) of the R1 primary fibroblasts at time points after IR were determined as described in Materials and Methods. 10 Gy IR-treated (black bars) or sham-irradiated (open bars) are shown. (TIF) [file pone.0077119.s004.tif]

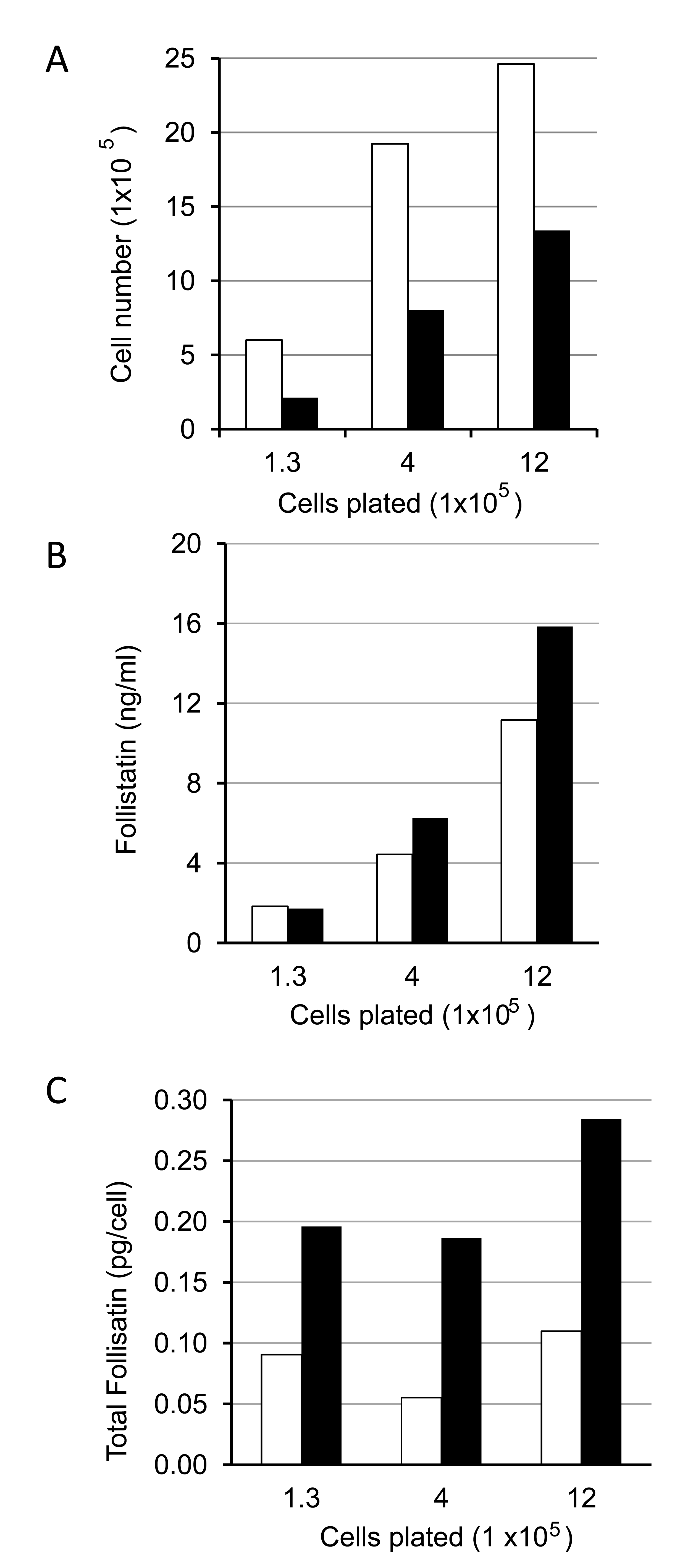

Supplement: Figure S5 — The effect of cell density on follistatin released into the medium after IR treatment. Cells were plated in 25 cm2 flasks at three densities and treated with 10 Gy IR (black bars) or sham irradiated (open bars). Twenty-four hours after IR treatment, cells were counted (A), follistatin was measured in the medium (B) as described in the Materials and methods, and normalized to cell number (C). (TIF) [file pone.0077119.s005.tif]
